# Stereotactic irradiation on linear accelerator - ultrasound versus MRI in choroidal melanoma volume calculation

**DOI:** 10.1186/s12886-022-02558-w

**Published:** 2022-08-05

**Authors:** Alena Furdova, Robert Furda, Miron Sramka, Martin Chorvath, Jan Rybar, Pavol Vesely, Jela Valaskova, Vladimir Siska

**Affiliations:** 1 Department of Ophthalmology, Faculty of Medicine, Hospital Ruzinov, Comenius University Bratislava, Ruzinovska 6, 826 06 Bratislava, Slovak Republic; 2 Department of Information Systems, Faculty of Management, Comenius University Bratislava, Odbojarov 10, 820 05 Bratislava, Slovak Republic; 3 Department of Stereotactic Radiosurgery, St. Elisabeth Cancer Institute and St. Elisabeth University College of Health and Social Work, Heydukova 10, 812 50 Bratislava, Slovak Republic; 4 Institute of Automation, Measurement and Applied Informatics, Faculty of Mechanical Engineering, Slovak University of Technology, Namestie slobody 17, 812 31 Bratislava, Slovakia

**Keywords:** Intraocular tumors, Uveal melanoma, Ultrasound, Magnetic resonance imaging, Stereotactic irradiation

## Abstract

**Background:**

Stereotactic irradiation is one of the treatment modalities for intraocular uveal melanoma. The study’s purpose was to describe the background of stereotactic one-day session radiosurgery, how the comparison in the difference between the tumor volume measured values from the magnetic resonance imaging (MRI) method and the ultrasound method was related to it, and which method was more precise to be used for tumor regression after irradiation.

**Methods:**

The group of 147 patients with choroidal melanoma was treated by stereotactic irradiation on the linear accelerator with a single dose of 35.0 Gy. During the standard treatment process the uveal melanoma volumes, needed for dose calculation, were obtained using MRI from the individual stereotactic planning scheme and by ultrasound from the ultrasound device. All volumes were statistically compared using the paired t-test, and for the visualization purpose, the Bland-Altman plot was used.

**Results:**

In the group of patients, it was 70 (47.6%) males and 77 (52.4%) females. The tumor volume median was from MRI equal to 0.44 cm^3^ and from ultrasound equal to 0.53 cm^3^. The difference between the ultrasound and the MRI volume measured values was statistically significant. However, the Bland-Altman plot clearly documents that the two methods are in agreement and can be used interchangeably. In most of the cases, the measured values of the ultrasound-calculated volume achieved slightly higher measured values.

**Conclusions:**

The calculation of the intraocular uveal tumor volume is a crucial part of the stereotactic irradiation treatment. The ultrasound volume measured values were in most of the cases higher than the measured values from the MRI. Although the methods are comparable and can be used interchangeably, we are recommending using the more precise MRI method not only during the treatment but also on later regular medical checks of tumor regression or progression.

## Background

In adults, the most common type of intraocular cancer is intraocular melanoma, which originates from uvea. The incidence of uveal melanoma in the United States was approximately 4.0 new cases per million population per year in the last statistical overview. In European countries, the incidence of intraocular melanoma reported a similar value. Specifically in Slovakia, the value, reported in the National Oncology Register, was similar to the surrounding countries [1, 2].

A prognostic indicator of intraocular melanoma is tumor volume [3]. Many diagnostic tools are currently used to diagnose primary uveal melanoma, for example, ultrasound, optical coherence tomography (OCT), computed tomography (CT), magnetic resonance imaging (MRI), and positron emission tomography along with the CT (PET/CT). The diagnosis of early-stage intraocular melanoma disease is extremely important in treatment. Today several irradiation techniques have been used as the preferred treatment method for patients with uveal melanoma, like brachytherapy, stereotactic radiotherapy, proton beam irradiation, Leksell Gama Knife [4–7].

A technique for delivering high-precision volume-staged stereotactic radiosurgery has been developed not only for intraocular tumors but also for treating large arteriovenous malformations by using the Leksell Gamma Knife Perfexion. The method uses the hybrid of combining the landmark registration and voxel-based 3D image registration to enable multiple staged partial-volume treatments [8]. Frameless stereotactic radiosurgery with the linear accelerator is known also for other tumor types but also for brain metastases therapy [9].

For uveal melanoma, in the last decades, one of the frequently used treatment possibilities is the stereotactic photon beam irradiation that can be performed in the form of the single-fraction stereotactic radiosurgery, for example, with the gamma knife, the linear accelerator, or the cyberknife [10–12]. In Slovakia, the procedure is performed using the linear accelerator as a one-day surgery with a single dose of 35.0 Gy. The image contrast fusion, enhanced by MRI with the patient’'s CT, is used to develop a three-dimensional treatment plan and to calculate the plan’s coordinates before irradiation. The single 35.0 Gy fraction is administered with high spatial accuracy by using the collimation system. The team of specialists includes one ophthalmologist, one neurosurgeon, one radiologist, and one medical physicist. They commonly create the individual planning scheme for the patient. The image fusion with contrast enhancement is suitable for precise specification of the affected anatomical structures around the tumor (lens, optic nerve, macula). Also, the fusion includes targeted volume differentiation of tumor mass, healthy tissue, and critical structures like intraocular lenses, optic chiasm, brainstem, eyelids, and optic nerves. Accurate planning is essential in determining the stereotactic coordinates of the radiation beam that will be applied to the targeted tumor mass defined by MRI parameters. Unwanted irradiation of critical structures can lead to visual acuity decline or to loss of visual acuity [13].

Ultrasound and MRI detection are crucial in the measurement of the tumor size to obtain its volume before the irradiation process but also after treatment patient’`s status. Not to forget, ultrasound is also significant in ophthalmology in defining the difference between the tumor mass and the intraocular bleeding or differentiating between primary or secondary retinal detachment. In practice, in uveal melanoma, the post-measurement volume calculation is using various methods. The volume measurement can be performed, for example, by using the ultrasonic 3D scanning system that operates in using the axial, back and forth half-rotation of the sector scanner with computer-assisted image analysis. The method is repeatable in significant correlation with other tumor measurement techniques that focus on, by MRI provided, measurement of the basal diameter for the tumor volume and prominence calculation [14].

The tumor volume in the eye globe is calculated using various mathematical formulas [15]. The formula employed by Guthoff is one of the standard formulas and means the volume calculation of the biconvex cross-sectional area of two spherical segments [16]. The calculation began to be used in the 1980s but does not come from the usual practice of ophthalmologists. Later, however, the following formula began to be used in calculating the tumor volume (TV): *“TV = π*/6 × (*largest basal diameter* × *width* × *prominence*)” by Gass. He hypothesized that the uveal melanoma grows ellipsoidal. In his studies, he included the volume calculated in this way in the calculation of the melanoma growth rate and compared the results obtained with histopathology and prognosis. Each intraocular melanoma was found to grow continuously, but the rate of growth itself varied between tumors in different patients [17].

A different model was invented to examine the growth rate using the exponential growth model with the same formula for tumor volume used by Gass [18]. The growth rate of the tumor was defined as the relative change in tumor size per unit of time. Also, faster-growing tumors developed earlier metastases and local radiation control fails during treatment [18].

In one more recent study, the calculated tumor volume assumed that the tumor is part of the spheroid of the intersecting sphere with the remarkably sophisticated formula. The non-elliptical baseline tumor area was calculated, which proved to be a more accurate prognostic indicator in predicting metastatic death after proton irradiation than the largest tumor diameter or calculated tumor volume In a similar study, the authors examined whether tumor volume, calculated according to the easy-to-use formula of the half-volume rotating ellipsoid and rotated about the y-axis, was a more reliable prognostic indicator of survival than the tumor diameter or the tumor prominence. Tumor diameter and tumor prominence data are used to calculate the volume of the rotating ellipsoid that rotates above the y-axis (prominence) following the formula: 4/3πa^2^b. The measured tumor prominence can be introduced into the formula in the position *b*, and the measured tumor diameter is divided by two and then introduced into the formula in the position *a*. The volume will be therefore calculated using the formula “4/3πa^2^b” which will be divided by the two to generate the result for the calculated tumor volume [19].

The study aims the purpose to describe the background of stereotactic one-day session radiosurgery, how the comparison of the difference between the tumor volume measured values from the MRI method and the ultrasound method is related to it, and which method is more precise to be used for tumor regression after irradiation.

## Methods

**Fig. 1:**
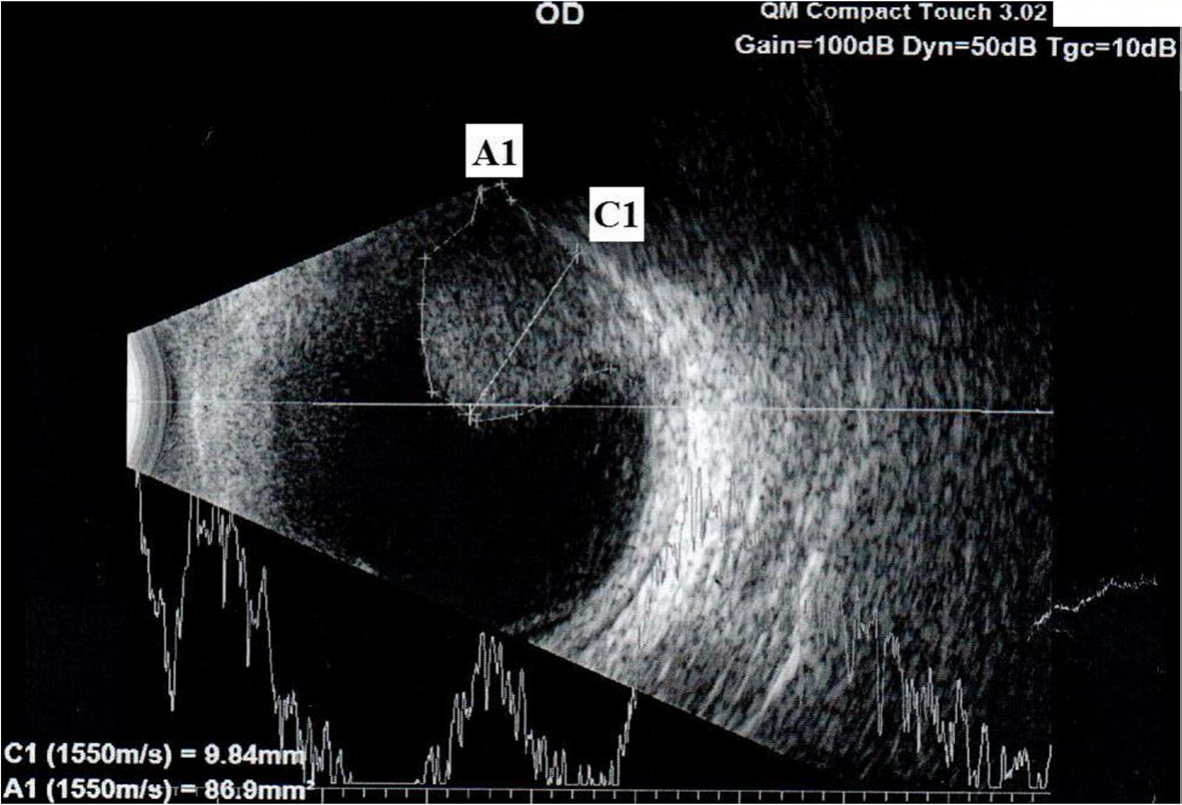
Examples of ultrasound findings in patient with uveal – choroidal melanoma. Left: large melanoma, C1 – elevation of the tumor (9.84 mm), A1 - area of the tumor (86.9 mm^2^), volume of the tumor was 0.8 cm^3^ (Source: Ultrasound Quantel Medical Compact Touch located at the workplace of the authors)

The data were collected from 147 patients in the period from 2009 to 2017. All patients in the T1-T3 stage of uveal melanoma underwent stereotactic irradiation on the linear accelerator LINAC in Slovakia. Before the intervention, the patients were investigated and measured by ultrasound (A-scan, B-scan with frequencies of 10 MHz) in the same center and by the same ophthalmologist during the period (Fig. 1). The formula used to obtain tumor volume was as follows *“TV = π*/6 × (*largest basal diameter* × *width* × *prominence*)”.

In each patient before the stereotactic intervention, the immobilization of the eye globe we achieved by mechanical fixation with sutures to the Leibinger stereotactic frame (Ceramic ring OSS - OSS head ring unit consisting of ceramic ring open OSS, 4 carbon-fiber head-fixing posts, locking bowl, toque wrench and fixing pins 40 mm, 50 mm, 60 mm). The sutures were placed through the conjunctiva under the insertions of the four direct extraocular muscles. The surgical preparation for intervention was performed 1 day before the irradiation in the Department of Ophthalmology. On the day of the irradiation, the stereotactic frame was attached to the patient’s head, and the sutures were tied to the stereotactic frame in the Department of Stereotactic Radiosurgery. After irradiation, the sutures, and stereotactic frame have been removed. The next morning, the patient underwent an ophthalmic examination by the slit lamp, a fundoscopy, and the measurement of intraocular pressure.

**Fig. 2:**
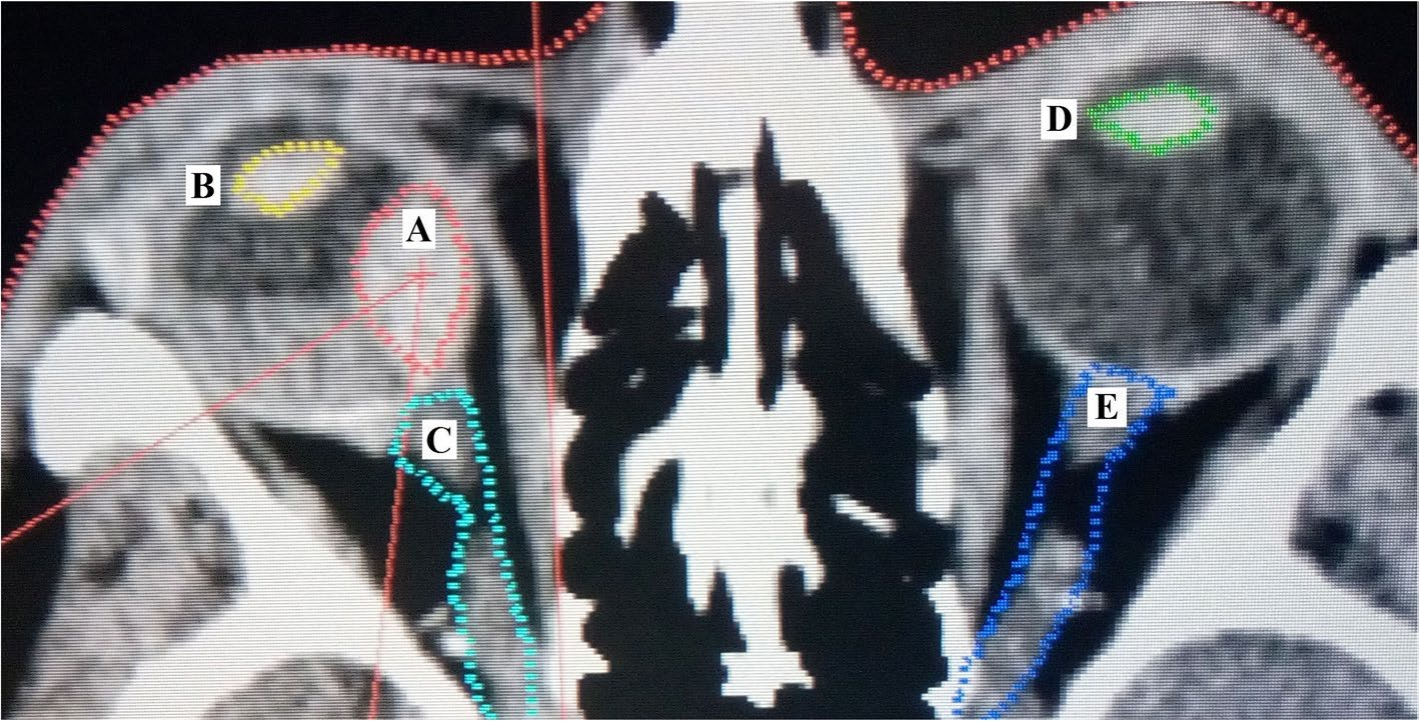
Stereotactic planning scheme - fused CT with MRI findings’ scans of the patient with uveal – choroidal melanoma at the day of stereotactic irradiation: **A** – tumor, **B** – lens of the irradiated eye globe, **C** - optic nerve of the eye, with uveal tumor, **D** - contralateral lens, **E** - contralateral optic nerve (Source: Embedded planning software of LINAC C 600 C/D Varian located at the workplace of the authors)

By default, the patient underwent the CT scan and the MRI with an already fixed eye globe to the stereotactic frame just before stereotactic irradiation on the linear accelerator. For tumor volume, the measured value from MRI, calculated according to tumor ROI (region of interest) and included in the irradiation protocol, and the MRI fusion with CT scans were used for the stereotactic planning scheme (Fig. 2). The planning of stereotactic treatment after the fusion is standardly optimized according to critical structures that include the lens, the optic nerve of the eyes, as well as the chiasm. The model C LINAC 600 C/D Varian, Aria system, Corvus ver. 6.2 Verification of the IMRT OmniPro with the frame of 6 MeV X was used as the linear accelerator. The therapeutic dose for stereotactic irradiation was 35.0 Gy, TDmin dose to the margin of the lesion ranged from 35.0 Gy to 38.0 Gy, and TDmax from 37.0 Gy to 60.0 Gy. The PTV (planning treatment volume) method was implemented for 99% isodose planning. The doses to critical structures were below 8.0 Gy for the optic nerve and the optic disc, and 10.0 Gy for the eyelids and anterior segment of the eye.

**Fig. 3:**
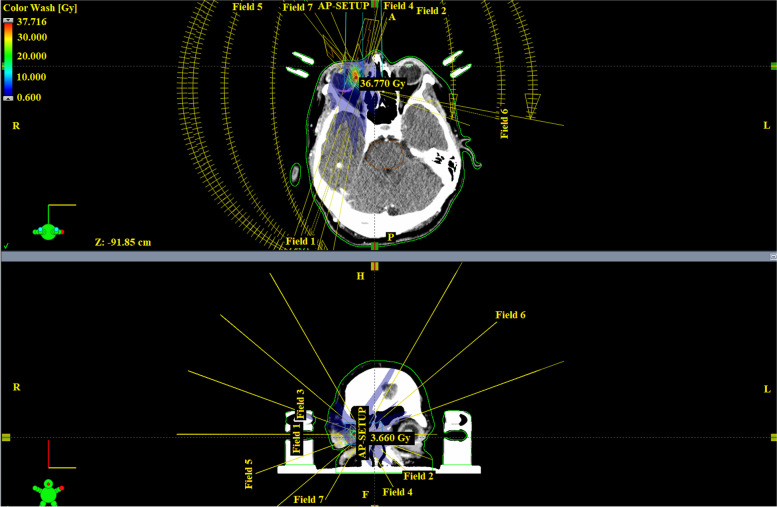
Stereotactic planning scheme for patient with uveal melanoma on linear accelerator (TD – 35.0 Gy) – part A (Source: Embedded planning software of Varian TrueBeam 2.7 located at the workplace of the authors)

**Fig. 4:**
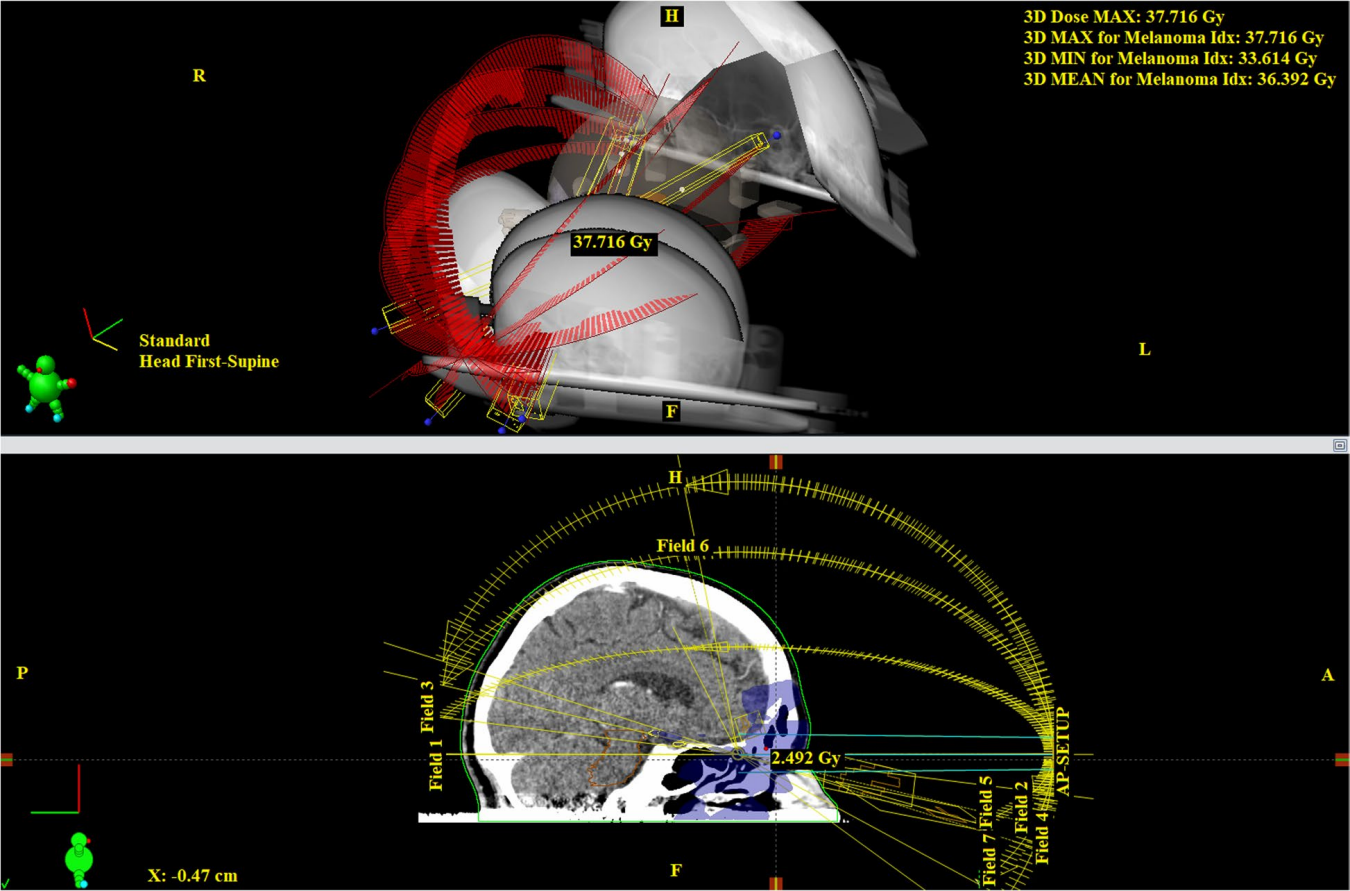
Stereotactic planning scheme for patient with uveal melanoma on linear accelerator (TD – 35.0 Gy) – part B (Source: Embedded planning software of Varian TrueBeam 2.7 located at the workplace of the authors)

Therefore, the data for evaluation and comparison included two sets of measured values: the ultrasound tumor volume measured values measured by an ophthalmologist using B-scan ultrasound, calculated using the embedded ultrasound measurements, and the MRI tumor volume measured values were taken from the patient’s irradiation protocol (Figs. 3 and 4). The time between the measurement of melanoma volume by the ophthalmologist with ultrasound and the stereotactic procedure was half a day, and the time interval between MRI-based measurement and procedure was approximately 1 month. The first post-operative control was done 3 months after irradiation, and the measured values were not statistically analyzed, because we did not expect differences between the measured values during irradiation and 3 months after irradiation. It was too short an interval to see any volume changes.

For the statistical evaluation, the paired sample t-test was used to demonstrate if the measured values of these sets can be the statistically significant difference found in the calculations. Additionally, the method of the Bland-Altman plot was used to visualize whether the two methods of the intraocular tumor volume calculation can be considered in agreement. For the purpose, the mean and standard deviation was derived, and the 95% ‘limits of agreement’ was calculated as the mean of the two measured values, minus, and plus, 1.96 standard deviations. The 95% ‘limits of agreement’ should contain the difference between both measuring systems for 95% of the measurement pairs.

## Results

In the group of 147 patients with choroidal melanoma, there were 97 cases in the right eye and 50 cases in the left eye, 70 males (47.6%) and 77 females (52.4%). The average age at the time of treatment was 62.05 years, 63.9 for males and 60.4 for females. On the basis of TNM staging, 77 melanomas were in stage T1 (52.4%), 44 in stage T2 (29.9%), and 26 in stage T3 (17.7%).

Results of ultrasound measurements documented the mean horizontal diameter of 10.55 mm (range 3.8 mm to 19.0 mm) and the mean vertical diameter of 6.75 mm (range 3.2 mm to 14.8 mm).

**Fig. 5:**
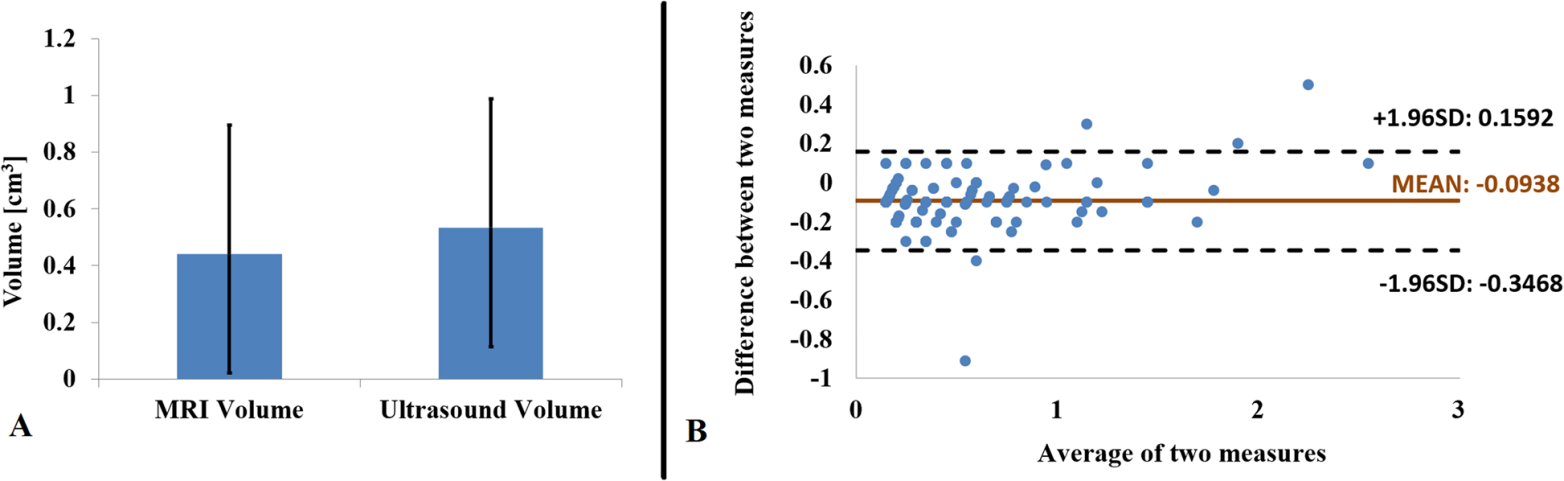
**A**: MRI Volume with median 0.44 (standard deviation = 0.46) and ultrasound volume with median 0.53 (standard deviation = 0.42), **B**: Bland-Altman plot of MRI and ultrasound methods (Source: MS Excel software located at the workplace of the authors)

The tumor volume obtained from the MRI had a median of 0.44 cm^3^ (range 0.09 cm^3^ to 2.6 cm^3^, the standard deviation was 0.46) and for ultrasound measurements, the calculations documented the median of 0.53 cm^3^ (range 0.1 cm^3^ to 2.5 cm^3^, the standard deviation was 0.42) (Fig. 5A).

By using paired sample t-test, applied on the set of ultrasound volume measured values and the set of MRI volume measured values:

- Median difference (MD) was 0.09, 95% confidence interval (CI) of this difference was from 0.0728 to 0.1149, and standard error of difference (SED) was 0.011.
- Two-tailed *P*-value is less than 0.0001 (exactly 3.35014E-15) so by conventional criteria, this difference is considering extremely statistically significant with t (146) = 8.811, and effect size r = 0.59.

**Fig. 6:**
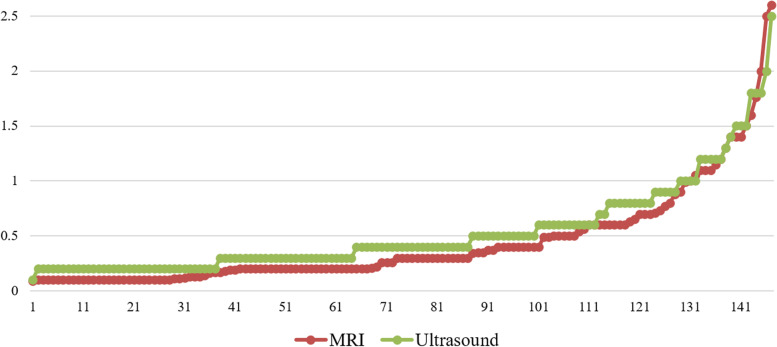
Group of 147 patients - measured values of MRI and ultrasound tumor volumes (cm^3^) that were sorted for better visualization (Source: MS Excel software located at the workplace of the authors)

According to our result, the difference between the set of the volume measured values calculated from the ultrasound measurement and the set of by MRI measured values was extremely statistically significant. But the Bland-Altman plot (Fig. 5B) visibly documents that the two methods are in agreement and can be used interchangeably. In most cases, the measured values of ultrasound calculated volume were higher than the measured values using MRI (Fig. 6). Even though the Bland-Altman plot presented demonstrated the strong concordance between the two techniques in certain conditions the secondary retinal detachment or subretinal fluid with bleedings (hemorrhage) can be evaluated as a part of tumor volume itself, and in certain very small places of the measured lesion volume, it can appear the difference between the volume - the measured value of the tumor itself.

## Discussion

Diagnosis and detection of primary uveal melanoma are primarily based on clinical examination by ultrasound and indirect ophthalmoscopy. However, MRI scans should also be performed to confirm the diagnosis and mark the patient for radiation therapy. Linear accelerator-based stereotactic irradiation is the right treatment for posterior uveal melanoma, is well-tolerated, and can be offered to medium-sized uveal melanoma patients [20]. Radiotherapy for uveal melanoma by CyberKnife system or stereotactic radiosurgery must quantify the reproducibility of the eye immobilization system. In all these modalities, it is necessary to use scans, x, y, and z to see the displacements of the lens, optic nerve insertion, and other structures to compare scans during the irradiation, and later on also to find the results of radiosurgery by regular check-ups [21].

Until now, there no multicenter trial to assess the efficacy and tumor volumetry of stereotactic radiosurgery has been performed. Also, the same is valid for gamma knife radiosurgery for intraocular melanoma. Results from more studies prove, for example, that irradiation techniques (by including the linear accelerator, proton beam irradiation, or gamma knife) have similar local results if compared to brachytherapy (typically, tumor control rate). The studies conclude that Gamma knife radiosurgery or linear accelerator stereotactic irradiation for uveal melanoma is the suitable alternatives in treating intraocular melanoma patients they were not indicated for brachytherapy [22, 23]. The majority of treated patients in our study were in stage T1, thus suitable for plaque brachytherapy, but this method is not available in Slovakia in the last decades.

In the case of the report by Jacobsen et al. 94-year-old man with a prior ocular history of age-related macular degeneration in both eyes was referred to an ophthalmology clinic for the routine-dilated fundus test. Contrast orbital MRI showed the sub-centimetric region of abnormal contrast enhancement extending into the immediately adjacent orbital fat, suspected of scleral invasion and small extrascleral extension of the lesion. Ultrasound was important to define the difference between intraocular bleeding and secondary retinal detachment. Both methods were important to provide the correct diagnosis before treatment. It is important to define the intraocular lesion, as metastatic uveal melanoma of the skin tissue can also occur, although it is exceedingly rare [24]. The optimal treatment for metastatic melanoma of the skin into the eyeball is not known but can include radiation therapy or systemic immunotherapy [25]. Survival after metastasis does not depend on the initial characteristics of the intraocular tumor. Even small tumors have a higher risk of metastasis after 10 years [26]. The potential spread after local destruction with endovitreal resection (removal) of the large uveal melanoma is also important for defining the volume of intraocular melanoma [27].

The use of contrast-enhanced ultrasound in the quantitative assessment of the response of intraocular melanoma to gamma-knife radiosurgery investigates whether changes in tumor vascularization precede the thickness reduction, which on average occurs at 12 radiosurgery after 12 months or later, as mentioned in the study of Venturini. The reduction of melanoma thickness occurred in 6 of the 10 patients, whereas the reduction in all quantitative parameters in all 10 patients [28]. Accurate tumor volume measured values can facilitate more reliable estimates of tumor regression or regrowth after treatment of globe-retaining choroidal melanomas and can be one of the valuable prognostic indicators after treatment [29]. But by stereotactic irradiation to observe a reduction of tumor volume, it is necessary to send regularly the patient to MRI after therapy. In our Center in the first 5 years interval after stereotactic irradiation, we send patients every 6 months for MRI and later at least once per year. Of course, ultrasound is also regularly performed by an ophthalmologist. In any case, ophthalmic ultrasonography performed by an ophthalmologist is still the most important tool to assess and monitor the progression of uveal melanomas and should be performed at certain intervals in each patient after stereotactic irradiation, until the end of his/her life. But last year and today we still do have problems keeping this interval due to COVID problems.

CT or MRI are considered to be the standard for the planning procedure also in stereotactic irradiation. The ultrasound measurement can be examiner dependent and less accurate with peripheral locations of the tumor. MRI was used to detect the extrascleral extension and in our study were these patients excluded from a one-day session of irradiation therapy. In our study, the patients with extrascleral extension were not indicated for stereotactic irradiation.

There is no consensus in the literature on how exactly the volume of the uveal melanoma should exactly be calculated due to the various forms of the uveal melanoma (dome versus mushroom shape). The rotating ellipsoid formula described by Richtig et al. was not reproducible on the larger data set. In our study authors, we provide the mean (median) horizontal diameter of the tumor and mean vertical diameter of the tumor (thickness, elevation of the lesion) but not the transverse diameter of the tumor, which in some cases can be bigger than the longitudinal section [19, 30].

To compare the results between the two measurements and evaluate the applicability of stereology and planimetry in computed tomography orbital volume measurements in the study, stereological measurements were superior to manual planimetry in terms of user effort and time spent [31]. Many studies suggested that other imaging modalities, such as MRI, are superior to ocular ultrasound in detecting extrascleral extension [32, 33].

In many countries, ophthalmologists do not use MRI to diagnose or observe post-irradiation changes in uveal tumor volume, they use only ultrasound. However, the ultrasound technique is routinely available in ophthalmology centers and is not so expensive, compared to MRI.

CT and MRI images are the base for stereotactic therapy in uveal melanoma. Why? It is due to safety and excellent local control. In our study, all patients underwent ocular B-scan ultrasound as the first step to assess the stage of the melanoma, and then every patient was sent to MRI to detect possible extrascleral extension. Our experience still confirms that ocular ultrasound is essential in patients with uveal melanoma. In patients in whom stereotactic irradiation was indicated based on the ultrasound findings, the MRI remains the next fundamental step for verification. In this treatment, we excluded the patients with tumor volume below 0.1 cm^3^ because the extremely small tumor volumes are not suitable for irradiation and cause problems with stereotactic planning.

The measured values of tumor volume, measured by ultrasound and MRI, were different, and the stereotactic irradiation was performed according to the MRI or CT image, but not ultrasound. Ultrasound is a common method for the ophthalmic oncologist, so MRI is necessary for planning, but follow-up and response should be judged by ultrasound, not frequent MRI in practice but in certain conditions, it is necessary to use MRI to evaluate the volume regression, because the interpretation of ultrasound image is difficult.

The findings from the MRI verification step, which can detect possible extraocular extension, were also examined by our neuro-radiologist to evaluate specific inflammation, vascular formation (e.g., feeding vessel), or movement artifacts. These findings cannot be generalized to every patient, and evaluation and radiation therapy should be planned on a case-by-case basis. Thus, an ultrasound must be performed by an experienced ophthalmologist in all patients with uveal melanoma. Additionally, each patient is unique, the measuring procedure and resources are often limited, and the ocular ultrasound should represent not only a unique imaging modality of choice in the search for extrascleral spreading in uveal melanoma but also a basic examination in onco-ophthalmology.

## Conclusions

The exact measured value of the tumor volume is important for calculating the irradiation planning scheme using irradiation techniques, and after treatment, this measured value can be a valuable prognostic indicator. The tumor volume calculation by MRI is one of the most crucial factors to indicate stereotactic radiosurgery treatment but also before ultrasound by evaluating the volume regression of the tumor after irradiation therapy.

In our study, the ultrasound volume measured values of the calculated tumor were higher than the MRI volume measured values. The reverse analysis of the measurements discovered that the ophthalmologist delimited the tumor differently in ultrasound than the MRI.

It would be appropriate to provide such a comparison with a much larger group of patients by different irradiation or radiosurgery techniques for uveal melanoma. Due to our results, the MRI picture is basic for future controls of the tumor reduction or, progression, and is better than ultrasound. We accept from practice that MRI volume measurements are more accurate than the results of ultrasound volumetric calculations for purposes in stereotactic planning schemes. Nevertheless, it should be emphasized that ophthalmic ultrasonography performed by the experienced ophthalmologist is still the most important aid in evaluating the development or reduction of the volume of intraocular uveal melanoma after treatment.

Anyway, we think that in patients after stereotactic radiosurgery tumor volume calculated from MRI is one of the most crucial factors to indicate stereotactic radiosurgery treatment but also to observe volume changes after irradiation, and ultrasound is not sufficient, even though it is cheaper and available in ophthalmology centers, it is necessary to send patients regularly to MRI examination after stereotactic radiosurgery due to intraocular uveal melanoma to see the progression or regression of uveal tumor mass.

## Data Availability

Data available on request from the corresponding author “alikafurdova@gmail.com”.

## Abbreviations

3D: Three-dimensional
CI: Confidence interval
CT: Computed tomography
LINAC: Linear (particle) accelerator
MD: Median difference
MRI: Magnetic resonance imaging
OCT: Optical coherence tomography
OSS: Open stereotactic system
PET/CT: Positron emission tomography along with a CT
PTV: Planning treatment volume
ROI: Region of interest
SED: Standard error of difference
TNM: The TNM classification of malignant tumors
TV: Tumor volume
